# Sulfated Polysaccharide Extracted from the Green Algae *Codium bernabei*: Physicochemical Characterization and Antioxidant, Anticoagulant and Antitumor Activity

**DOI:** 10.3390/md20070458

**Published:** 2022-07-15

**Authors:** Fabian A. Figueroa, Roberto T. Abdala-Díaz, Claudia Pérez, Virginia Casas-Arrojo, Aleksandra Nesic, Cecilia Tapia, Carla Durán, Oscar Valdes, Carolina Parra, Gastón Bravo-Arrepol, Luis Soto, José Becerra, Gustavo Cabrera-Barjas

**Affiliations:** 1 Laboratorio de Química de Productos Naturales, Departamento de Botánica, Facultad de Ciencias Naturales y Oceanográficas, Universidad de Concepción, Casilla 160-C, Concepción 4030000, Chile; fafigueroa@udec.cl (F.A.F.); claudiaperez@udec.cl (C.P.); a.nesic@udt.cl (A.N.); luisoto@udec.cl (L.S.); jbecerra@udec.cl (J.B.); 2 Unidad de Desarrollo Tecnológico (UDT), Universidad de Concepción, Avda. Cordillera No. 2634, Parque Industrial Coronel, Coronel 4191996, Chile; gastonbravo@udec.cl; 3 Departamento de Ecología, Facultad de Ciencias, Instituto de Biotecnología y Desarrollo Azul (IBYDA), Universidad de Málaga, Campus de Teatinos s/n, 29071 Málaga, Spain; virginia@uma.es; 4 Vinca Institute of Nuclear Sciences—National Institute of the Republic of Serbia, University of Belgrade, 12–14 Mike Petrovića Street, 11000 Belgrade, Serbia; 5 Laboratorio de Especialidad Clínica Dávila-OMESA, Recoleta 464, Recoleta, Santiago 8431657, Chile; cvtapiap@gmail.com (C.T.); cduran@vidaintegra.cl (C.D.); 6 Centro de Investigación de Estudios Avanzados del Maule (CIEAM), Vicerrectoría de Investigación y Postgrado, Universidad Católica del Maule, Talca 3480005, Chile; ovaldes@ucm.cl; 7 Laboratorio de Recursos Renovables, Centro de Biotecnología, Barrio Universitario s/n, Universidad de Concepción, Concepción 4030000, Chile; roparra@udec.cl; 8 Centro Nacional de Excelencia Para la Industria de la Madera (CENAMAD), Pontificia Universidad Católica de Chile, Vicuña Mackenna 4860, Santiago 7820436, Chile; 9 Centro de Investigación de Polímeros Avanzados, Edificio Laboratorio (CIPA), Avda. Collao 1202, Concepción 4051381, Chile

**Keywords:** *Codium bernabei*, sulfated galactans, polysaccharides, anticoagulant activity, antioxidant capacity, cytotoxic effect

## Abstract

*Codium bernabei* is a green alga that grows on Chilean coasts. The composition of its structural polysaccharides is still unknown. Hence, the aim of this work is to isolate and characterize the hot water extracted polysaccharide fractions. For this purpose, the water extracts were further precipitated in alcohol (TPs) and acid media (APs), respectively. Both fractions were characterized using different physicochemical techniques such as GC-MS, GPC, FTIR, TGA, and SEM. It is confirmed that the extracted fractions are mainly made of sulfated galactan unit, with a degree of sulfation of 19.3% (TPs) and 17.4% (ATs) and a protein content of 3.5% in APs and 15.6% in TPs. Other neutral sugars such as xylose, glucose, galactose, fucose, mannose, and arabinose were found in a molar ratio (0.05:0.6:1.0:0.02:0.14:0.11) for TPs and (0.05:0.31:1.0:0.03:0.1:0.13) for ATs. The molecular weight of the polysaccharide samples was lower than 20 kDa. Both polysaccharides were thermally stable (Tonset > 190 °C) and showed antioxidant activity according to the ABTS^•+^ and DPPH tests, where TPs fractions had higher scavenging activity (35%) compared to the APs fractions. The PT and APTTS assays were used to measure the anticoagulant activity of the polysaccharide fractions. In general, the PT activity of the TPs and APs was not different from normal plasma values. The exception was the TPs treatment at 1000 µg mL^−1^ concentration. The APTTS test revealed that clotting time for both polysaccharides was prolonged regarding normal values at 1000 µg mL^−1^. Finally, the antitumor test in colorectal carcinoma (HTC-116) cell line, breast cancer (MCF-7) and human leukemia (HL-60) cell lines showed the cytotoxic effect of TPs and APs. Those results suggest the potential biotechnological application of sulfate galactan polysaccharides isolated from a Chilean marine resource.

## 1. Introduction

Green algae or Chlorophytas present a significant group of marine algae, which are a source of polysaccharides. The *Bryopsidales* family of green algae (including the genera Bryopsis, Codium, and Derbesia) have existed for millions of years [1]. However, cell wall polysaccharides from green algae have been less studied than those from both the red (i.e., carrageenan and agarans) and brown algae (i.e., alginate, fucoidan, and laminaran), respectively. This fact is probably related to their lower industrial applications [2,3,4]. Nevertheless, in recent years the study of sulfated polysaccharides from species of the Codium genus has gained increasing interest from the scientific community. It is mainly due to the structural diversity of polysaccharides contained in Codium family algae and their interesting physicochemical and biological properties [5,6,7]. This family of green algae has the advantage of being widely distributed around the world, comprising around 150 species that grow from temperate to tropical seas [8].

In particular, green algae synthesize various types of sulfated homo- and heteropolysaccharides [9]. The striking dissimilarities in those polysaccharide bioactivities are probably related to their different chemical structure [10]. However, it is possible to find common structural factors in the numerous biological activities demonstrated for sulfated polysaccharides. For instance, the monomeric composition of heteroglycans is composed mainly of fucose, xylose, glucose, galactose, uronic acid monosaccharides, and sulfate groups [11]. Moreover, the most bioactive and promising candidates are sulfated polysaccharides. They show a variety of glycosidic linkages, leading to branched structures, and attached sulfate groups with different spacial distributions [12].

In this line, it has been reported that species from the Codium family produce sulfated galactans that show heterogeneity and structural complexity [13]. It has been described that the polysaccharide bioactivity is influenced by the degree of sulfation, the position of these groups in the sugar ring, the spatial structure, the molecular weight, and the monosaccharide composition [14]. The Codium polysaccharides exhibit several biological activities, including anticoagulant, immunomodulatory, antitumor, antioxidant, anti-inflammatory, neuroprotective, and hypoglycemic activity [15,16,17,18,19,20,21]. For example, *Codium fragile* and *Codium vermilara* can provide water-soluble sulfated arabinogalactans with anticoagulant activity [5,22]. Additionally, Kolsi et al. [23] demonstrate that the polysaccharides extracted from *C. fragile* possess antioxidant activity and can reduce oxidative stress preventing the generation of free radicals and subsequently avoiding the development of atherosclerosis. Other authors have shown that sulfated galactans polysaccharides isolated from *C. cylindricum* have antiangiogenic activity [24]. Additionally, Lee et al. [25] showed that arabinogalactans isolated from *C. fragile* have immunostimulatory effects through the activation of macrophages, inducing the expression of cytokines IL-1β, IL-6, IL-10, and TNF-α. Additionally, they showed that it can elicit immunity against lung cancer in mice [19]. In this line, Surayot et al. [26] demonstrated that sulfated polysaccharides of *C. fragile* activate NK cells inducing antitumor activity improvement. Those results make them interesting compounds with potential applications in pharmacological, nutraceutical, and food industries [27].

According to the author’s knowledge, this will be the first approach that has studied the composition of *Codium bernabei* green algae polysaccharides. For this purpose, the hot water extracts were precipitated by alcohol and precipitated selectively with N-cetylpyridinium bromide (Cetavlon). Both fractions were characterized by several techniques, such as gas chromatography-mass spectrometry (GC-MS), Fourier transform infrared spectroscopy (FTIR), gel permeation chromatography (GPC), elemental analysis, thermogravimetric properties (TGA-DSC), and scanning electron microscopy (SEM). Moreover, the biological activity of polysaccharides fractions was determined through their antioxidant, anticoagulant, and antitumor activity test.

## 2. Results and Discussion

### 2.1. Polysaccharide Characterization

#### 2.1.1. GC-MS Sugar Analysis

**Figure 1:**
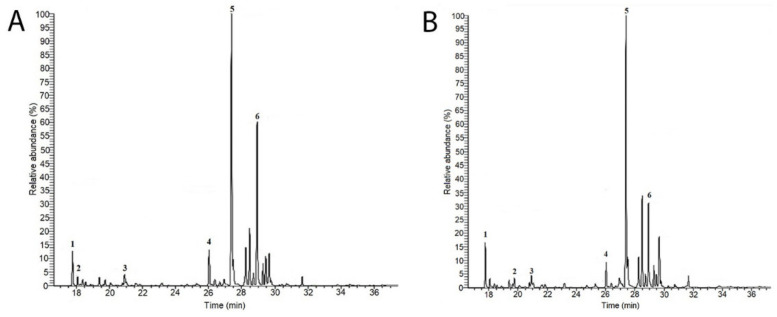
GC-MS chromatograms of the TPs (**A**) and APs (**B**) polysaccharides fractions isolated from *C. bernabei*. Monosaccharide identified are as follows: 1 Arabinose; 2 Fucose; 3 Xylose; 4 Mannose; 5 Galactose; 6 Glcucose.

The monosaccharide compositions of *C. bernabei* polysaccharides extracted in hot water and precipitated in alcohol (TPS sample) and in acid media (Aps sample) were determined by GC-MS of their trimethylsilyl (TMS) derivatives (Figure 1). The identification of monosaccharide derivatives was carried out by comparing their retention times and by their characteristic GC-MS fragmentation patterns. The monosaccharide and protein contents, degree of sulfation, and molecular weight for each polysaccharide are summarized in Table 1. The GC-MS spectra showed the presence of six major sugars in the TPs and APs samples. They were identified as arabinose (Arb), fucose (Fuc), xylose (Xyl), mannose (Mann), galactose (Gal), and glucose (Glc).

It is observed from Table 1 that both samples were mainly constituted by Gal and Glc monosaccharides, which represented more than 80% of the total sugar, respectively. It is known that the genus Codium is very diverse, containing approximately 150 species with a variety of morphology around the world [7]. In this context, also included in Table 1 are the chemical composition of other species of the genus Codium, such as *C. decorticatum*, *C. fragile*, and *C. vermilara*, for comparison.

The sugar compositions of the extracted samples are similar to that reported for *C. vermilara* and *C. fragile* [5,13,28,29]. In both studies, Gal was the main component (>50%) of the polysaccharides. However, other studies revealed that instead of galactose, arabinose represented the main sugar residue of the *C. decorticatum* polysaccharides, with a proportion of up to 48% [30,31,32]. On another side, the degree of sulfation in TPS is slightly higher than in APs, but they are lower than reported for *C. decorticatum* and *C. vermilara,* and higher than that of *C. fragile* [13,28,29,30,31,32]. It is known that this parameter is one of the main chemical features that determine the biological activity of sulfated polysaccharides. The protein content of APs is very low regarding the TPs polysaccharide, which could be due to the used preparation method. In the first case (APs fractions), the polysaccharide isolation involves opposite charge precipitation and solvent precipitation in the second case (TPs fractions). This fact could also account for the higher content of galactose units on the APs structure (61 mol%), which indicates it is a sulfate galactan polysaccharide. Furthermore, the molecular weight of APs is slightly lower than of TPs, but it has a better polydispersion index (1.6). The higher weight average (Mw) and polydispersity index (PI) of TPs could be due to the presence of proteins and other polysaccharide residues (Glu 31 mol%) in the sample.

The observed variations in the chemical composition of Codium species may be related to the influence of several exogenous and endogenous factors, such as temperature, light intensity, daylight length, the concentration of nutrients, growth, morphological change, reproduction, etc. [33]. Finally, it can be assumed that the sugar content of each component can be attributed to the species, location, collection season, and extraction conditions. All of these properties could be directly related to the biological activities of sulfated polysaccharides.

#### 2.1.2. Fourier Transform Infrared Spectroscopy (FTIR)

**Figure 2:**
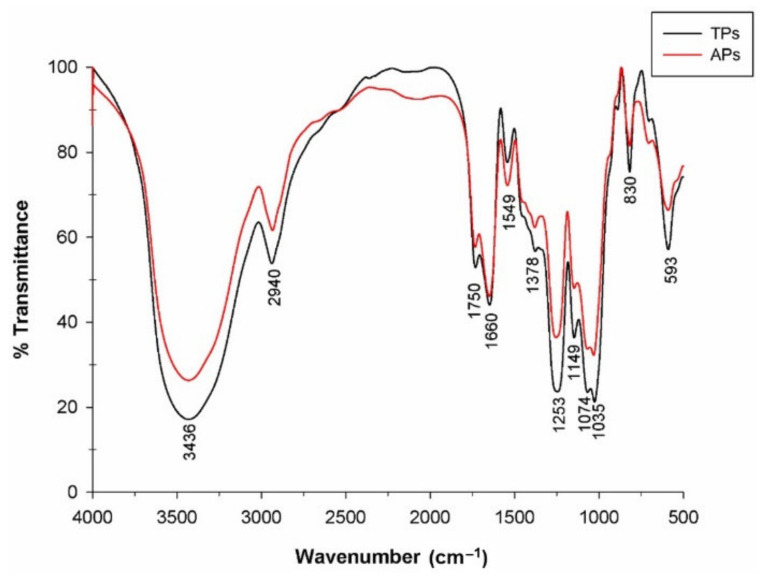
FTIR spectra of the polysaccharide fractions isolated from *C. bernabei* macroalgae.

The FTIR spectra were registered to identify the main functional groups of polysaccharide fractions isolated from the *C. bernabei* green macroalgae, and the results are presented in Figure 2.

Analyzing the spectra between 400 and 4000 cm^−1^ from Figure 2, similar bands to others from macroalgae polysaccharides could be observed. The band at 3438 cm^−1^ appeared as a broad stretching vibration that corresponds to the O–H group, and the small signal at 2941 cm^−1^ was attributed to C–H stretching and bending vibrations [34,35]. The absorption bands at 1750, 1660, and 1549 cm^−1^ were attributed to the stretching vibration of the C = O group [35,36]. According to the literature, the presence of sulfate groups for different Codium species was reported around 1229–1253 cm^−1^ [37,38,39]. In the spectral region between 1200 and 800 cm^−1^, known as the fingerprint region of polysaccharides, the presence of sulfate groups was identified in both polysaccharides as stretching vibration at 1258 cm^−1^, which was attributed to the S = O group and individually to sulfate esters [40,41,42]. In addition, the bands at 1149–1035 cm^−1^ corresponded to the C–O–C, C–OH, and C–C vibrations of the glycosidic ring [19]. The band at 830 cm^−1^ indicated the vibration attributed to sulfation in C2 of Gal units [43].

#### 2.1.3. Thermogravimetric Properties (TGA-DSC)

**Figure 3:**
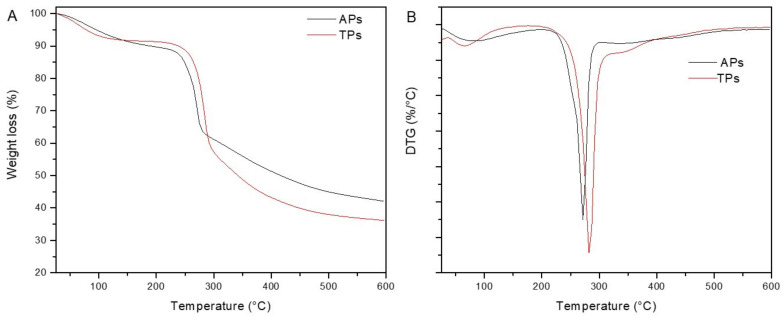
TGA (**A**) and DTG (**B**) curves of the polysaccharide fractions isolated from *C. bernabei* macroalgae.

The thermal characterization of a sample is an important step in the study of thermal transitions, which can reveal important information about appropriate temperature ranges compatible with polysaccharide processing [44]. Thermal analysis was performed in the temperature range from 30 to 600 °C under an N_2_ atmosphere to study the thermal degradation of the *C. bernabei* polysaccharides. The comparative TGA-DTG curves of APs and TPs samples are presented in Figure 3, and the results are summarized in Table 2.

According to the results, the thermal degradation of both polysaccharides was characterized by two different stages (Figure 3B). The first weight loss was associated with water evaporation up to 100 °C absorbed by the polysaccharides, which was 8.2% in TPs and 8.1 in APs. The second and most important degradation stage started at 208.6 °C and 196.8 °C for APs and TPs, having associated weight losses of 37.2% and 52.7%, respectively (Figure 3A). This degradation step was associated with the degradation of the C–O and C–C bonds in the ring units. The obtained results indicated that APs was more thermally stable than TPs, which could be associated with their different chemical composition. The difference in proteins and mineral content and degree of sulfation could account for higher weight losses associated with TPs. Moreover, according to the char residue values, it could be said that the APs sample contained more mineral char than the TPs sample.

In this regard, Parikh and Madanwar [45] described that the thermal degradation of polysaccharides is defined as a process with the following steps: (i) desorption of physically absorbed water, (ii) removal of structural water (dehydration reactions), (iii) depolymerization accompanied by the breakdown of the C–O and C–C bonds in the ring units, resulting in the evolution of CO, CO_2,_ and H_2_O, (iv) and the formation of aromatic and graphitic polynuclear carbon in the structures. In this study, different trends in the shapes of the TPs and APs decomposition curves were observed, mainly due to the difference in the composition of each polysaccharide [46].

Previous studies on the mechanisms of thermal degradation of *Ulva* sp. polysaccharides carried out by Alves et al. [47] observed that Ulvan sulfated polysaccharides were thermally stable until 200 °C. Moreover, Rodríguez-Jasso et al. [48] hydrolyzed fucoidans extracted from the brown seaweed *Fucus vesiculosus* that were thermally stable up to 215 °C; their devolatilization occurred between 215–490 °C, and their final degradation appeared around 470 °C. Those results are in agreement with the present work, confirming the sulfated polysaccharidic nature of the extracted polysaccharides.

#### 2.1.4. SEM Analysis

**Figure 4:**
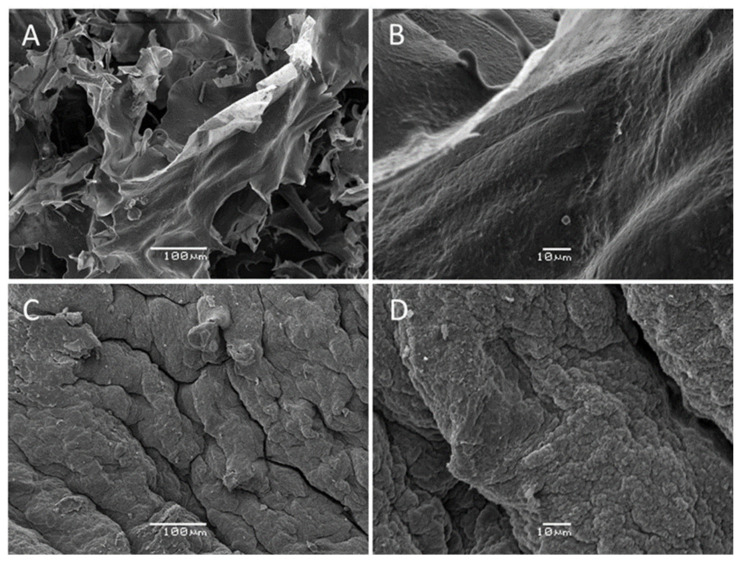
Scanning electron microscopy (SEM) images of TPs (**A**,**B**) and APs (**C**,**D**) polysaccharide fractions isolated from *Codium bernabei*.

The SEM technique allows for the study of the morphology of Codium extracted polysaccharides, and the obtained micrographs are presented in Figure 4.

In Figure 4, a different morphological structure was observed for APs and TPs fractions. The TPs sample (Figure 4A,B) had the appearance of a fibrous sheet-like structure, whereas in the APs (Figure 4C,D), a highly rough surface was noticed. Such a topological difference could be due to the methodology used to prepare them. In the first case, cold alcohol precipitation was used, which could allow for preserving the complex supramolecular structure of precipitated polysaccharides. This precipitate might contain some amount of other neutral polysaccharides, proteins, and minerals that contributed to stabilize an original fibrillar structure. This result is in alignment with the obtained higher neutral sugar content (glucose), higher protein content, and higher Mw for the TPs sample. Conversely, the natural fibrillar order was broken due to the charge-based precipitation used for APs sample isolation. Consequently, a non-ordered structure with low protein content was obtained and produced a rough surface appearance. These results were in agreement with previous studies on sulfated polysaccharides from seaweed that have concluded that the molecular weight and chemical composition of sulfated polysaccharides may influence their surface morphologies [49,50].

### 2.2. Biological Activity

#### 2.2.1. Antioxidant Activity

**Figure 5:**
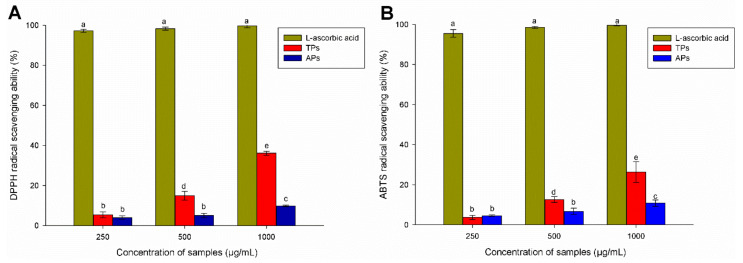
Antioxidant activity of polysaccharides extracted from *C. bernabei*. (**A**) DPPH radical scavenging (%) activity; (**B**) ABTS^•+^ radical scavenging activity (%). Ascorbic acid was used as Control. Values are presented as mean ± SD (*n* = 4). The different letters above the bars of each parameter are significantly different at *p* < 0.05 (*Tukey’s* test).

The ABTS radical (2.2′-Azino-Bis (3-ethylbenzothiazoline-6-sulfonic acid) and DPPH radical (2,2-diphenylpicrylhydrazyl) assays have been widely applied to evaluate natural compounds free radical scavenging activities in both lipophilic and hydrophilic samples [51]. In the case of DPPH, the antiradical capacity of the samples was obtained from the decrease in the absorbance of the DPPH radical at 517 nm [52]. Specifically, the reduction in the absorbance of the DPPH radical is due to the hydrogen atom transferred from an H donor leading to the disappearance of the visible band in DPPH [53]. On the contrary, the ABTS assay is a quick method for determining the antioxidant activity of hydrogen-donating compounds to aqueous phase radicals and the chain-breaking antioxidants of lipid peroxyl radicals [54]. Figure 5 shows the antioxidant activity of polysaccharides extracted from *C. bernabei*, specifically the DPPH radical scavenging (%) and ABTS^•+^ radical scavenging (%). Both cases used a positive control of ascorbic acid at 10 µg/mL.

The radical scavenging effect of TPs and APs was in a range from 0 to 40% over the concentration of extracted polysaccharides between 250 and 1000 µg mL^−1^. From Figure 5A, it could be seen that the DPPH radical scavenging activity of TPs was higher than that obtained by the APs. The DPPH radical scavenging activity increases in a concentration-dependent manner. The highest scavenging effect of 36.13% and 10% is observed at a concentration of 1000 µg/mL for the TPs and APs samples, respectively. However, the antioxidant activities are significantly lower when compared to the value for ascorbic acid, which was used as a positive control at the same concentration (99.63% at 1000 µg/mL). Comparing the obtained results with the literature, it can be noticed that the DPPH antioxidant activity of the TPs fractions is higher than for the several sulphated polysaccharides at the same or higher tested concentrations. For example, the sulfated polysaccharides of the *Ascophyllum nodosum* (Mw 27.96 KDa, sulfate content 28.60%, fucose content 30.14%, galactose content 5.01%, glucose content 5.01%, xylose content 18.24%, and mannose content 11.52%) had DPPH inhibition two times lower (16% at 3 mg/mL) than that reported for TPs in this work [55]. Fucoidan with unknown molecular weight (sulfate content 21.2%, fucose 76.8 mol%, galactose 23.2 mol%, total phenolics 5.6%) extracted from *F. vesiculosus* had 23% scavenging of DPPH at 1 mg mL^−1^) [56].

On the other hand, Figure 5B shows the ABTS^•+^ radical scavenging activity of the TPs and APs. The same trend and similar antioxidant values in antioxidant activity were obtained from this assay. The results indicated that the scavenging ability was associated with increased TPs concentration, reaching around 28% at the highest concentration. In this case, the positive control showed a significantly higher antioxidant activity.

Overall, the results show that TPs has higher radical scavenger activity than APs. It is known that polysaccharides show antioxidant activities, but they are much lower than displayed by other natural compounds (e.g., polyphenols). Several authors point out that polysaccharides do not always exist singly but conjugate with other components, such as amino acids, protein, lipids, and nucleic acid residues. Sometimes, the polysaccharide conjugates act as a whole in isolation, and such conjugates can enhance antioxidant activity [55]. In this work, the TPs fraction has a 15.6% protein content, whereas it is 3.5% in ATs. These differences could partially account for the observed difference in antioxidant activity. However, when concentration increases, it could not explain the exponential growth in antioxidant activity observed in the TPs but not in the ATs fraction. In this sense, TPs fractions have a slightly higher degree of sulfation, similar Mw, and lower fucose, galactose, and xylose content than ATs. It may be possible that the combination of all those structural factors acts synergistically. Previous results published by other authors demonstrated that the antioxidant activities of polysaccharides might be closely related to the higher degree of sulfation and lowest molecular weight (~21.4 KDa) but negatively correlated with the fucose, galactose, and xylose content [56,57,58].

#### 2.2.2. Anticoagulant Activity

The anticoagulant activity of the polysaccharides isolated from *Codium bernabei* was evaluated by thromboplastin time (APTT) and prothrombine time (PT) assays, and the results are summarized in Table 3.

In general, the PT activity of the TPs and APs was not statistically different (*p* < 0.001) from normal plasma values. The exception was the TPs treatment at 1000 µg mL^−1^ concentration, which showed a maximum prolongation of coagulation time for 6.9 s (for 1.6 fold time). The lack of significant effect on PT suggests that the extracted polysaccharide fractions do not inhibit the extrinsic pathway of coagulation. On the other hand, the APTTS test revealed that the clotting time for both polysaccharides was significantly (*p* < 0.001) prolonged regarding normal values at 1000 µg mL^−1^. The prolongation of APTT suggests the inhibition of the intrinsic and/or common pathways of coagulation. According to these results, TPs showed increased anticoagulant activity, reporting a concentration of 100 ug mL^−1^ and an increased clotting time of 20.1 s compared to the plasma control, on the opposite to APs (Table 2).

These results are similar to those reported by Chagas et al. [59], where it was demonstrated that at a concentration of 100 µg mL^−1^, the sulfated polysaccharide from red algae *Gelidiella acerosa* (degree of sulfation 0.69 and Mw = 248.8 kDa) had a prolonged clotting time in 2.1 times. The sulfated polysaccharide fraction from red algae *G. acerosa* (sulfate content 5–42%, total sugar content 2–24%) had a similar range of APTT values at 1 mg/mL [60]. On the other side, fucoidan from brown seaweed *Fucus vesiculosus* (sulfate content 27%, Mw = 735 kDa, fucose content 73.5%, glucose content 11.8%, galactose content 3.7%, xylose content 6.6%, mannose content 0.2%, and arabinose content 0.2%) prolonged the APTT time for 2.5 folds at concentration 3.2 µg/mL, when compared to the control [61]. On the other side, sulfated arabinans from *Codium vermilara* (sulfate content 44–54%) exhibited less or no anticoagulant activity [60]. Obviously, the chemical structure of sulfated polysaccharides has a significant role in the anticoagulant activity. It was demonstrated before that the anticoagulant activity of sulfated polysaccharides from Codium genes depended on the nature of the sugar residue, the position of sulfation, and the content of sulfation [61,62,63,64]. In addition, polysaccharides of higher molecular weight, higher sulfate content, and lower sugar content (Mw = 60.4 kDa, sulfate content = 35.6%, sugar content = 94.3%) extracted from Codium showed higher anticoagulant activities than polysaccharides with low molecular weight, sulfate, and sugar content (Mw = 3.36 kDa, sulfate content = 10.3, sugar content = 85.4%) [49]. However, Shanmugam and Mody showed that sulfated polysaccharides with molecular weight between 50 and 100 kDa could have good anticoagulant activity, whereas sulfated polysaccharides with molecular weight higher than 850 kDa exhibit low anticoagulant activity [62]. This fact is corroborated by findings in this paper, where it showed that TPs and Aps samples of small molecular weight (17.9 and 14.9 kDa, respectively) and intermediate degree of sulfation (19.4% and 17.2%, respectively) have a low effect on the PT test results and a moderate effect on the APTT results.

Anticoagulant activity has been previously described for sulfated polysaccharides, making them promising sources for the production of anticoagulant drugs. Adrien et al. [65] reported the anticoagulant activity of sulfated polysaccharide fractions from *Ulva rigida* macroalga, which was comparable to commercial anticoagulants such as heparin and Lovenox^®^. On the other hand, He M. et al. [66] demonstrated that the sulfated polysaccharide from the green alga *Cladophora oligoclada* significantly inhibited the activities of all intrinsic coagulation factors but selectively inhibited the common coagulation factors. In addition, the sulfated polysaccharide strongly stimulated thrombin inhibition by potentiating antithrombin-III (AT-III) or heparin cofactor-II and extensively promoted AT-III-mediated inhibition of factor Xa [66].

Sulfated polysaccharides could be used to produce anticoagulants as a replacement for heparin. Similar to other algae products, they could potentially find applications in the pharmaceutical industry to treat patients with cardiac pathology (unstable angina or infarction), catheterization of patients, deep vein thrombosis, and others.

#### 2.2.3. Cell Viability in Tumor Lines

**Figure 6:**
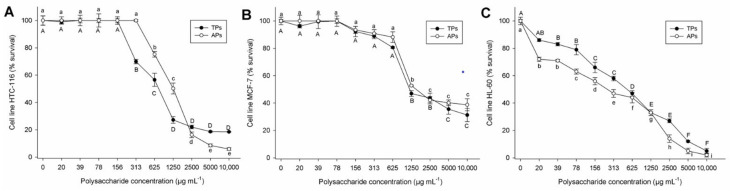
Cell viability (% survival) exposed to different concentrations of polysaccharide fractions from *Codium bernabei*. (**A**) Human colon cancer cell line (HTC-116), (**B**) Breast cancer cell line (MCF-7), (**C**) Human leukaemia cells (HL-60). The same letter indicate no significant difference at *p* < 0.05 (*Tukey’s* test).

In the last decade, incidence and mortality rates increased for various types of cancers, including colon cancer, breast cancer, and human leukaemia. In this context, algae polysaccharides have become interesting potential candidates for antitumor therapies. This study reports for the first time the cytotoxicity of polysaccharides extracted from *C. bernabei* on tumor cell lines. The colon cancer (HCT-116), breast cancer (MCF-7), and human leukaemia (HL-60) cells exposed to different polysaccharide concentrations for 72 h showed a dose-response effect and was observed a significant cytotoxic effect (*p* < 0.01) (Figure 6A–C).

In this study, in the HCT-116 cell line, the TPs and APs showed 50% inhibitory concentration (IC_50_) values of 760 µg mL^−1^ and 1370 µg mL^−1^, respectively (Figure 6A). Recently, sulfated polysaccharides from the brown algae *Colpomenia sinuosa* (unknown Mw, sulfate content 18.8%, molar % 67.4, 5.94, 3.58, 2.62, 5.45, and 3.4 for fucose, galactose, mannose, xylose, glucosa, and arabinose, respectively), at a concentration of 750 µg mL^−1^, showed a cell inhibition ability of 45% on HCT-116 cell line [67]. Conversely, sulfated polysaccharides (ScF2) extracted from the brown seaweed *Saccharina cichorioides* are not toxic against HCT-116 cells at doses up to 800 µg mL^−1^ [68]. Additionally, the IC_50_ reported in the MCF-7 line, when incubated at increasing concentrations of TPs and Aps, was 1118 µg mL^−1^ and 1952 µg mL^−1^, respectively (Figure 6B). Finally, in the HL-60 cell line, the TPs and APs showed IC_50_ of 540 µg mL^−1^ and 260 µg mL^−1^, respectively (Figure 6C). In a previous report, the sulfated polysaccharides of the brown seaweed *Laminaria ochroleuca* obtained from N-methylpyridinium bromide (Cetavlon) precipitation showed cytotoxic activity in human colon cancer cell line HTC-116 (IC_50_ = 440 µg mL^−1^) and breast cancer cell line MCF-7 (IC_50_ = 8320 µg mL^−1^) [69]. These results showed higher cytotoxicity than the extracted polysaccharides in the HTC line, but it was less active than TPs and APs in MFC cell lines.

According to these results, TPs showed low cytotoxicity on HCT-116 and MCF-7 cell lines when compared to APs sample. In contrast, the APs showed low cytotoxicity on HL-60 cell lines. Hence, it can be concluded that the antiproliferative effect of sulfated polysaccharides depends on line cell type. These results implied that extracted polysaccharides could exhibit selective cytotoxicity in tumor cells, and that could be good candidates for further investigation.

## 3. Materials and Methods

The *C. bernabei* specimens were collected in the rocky mid intertidal zone of Cocholgue, Biobío Region, Sea of Chile (36°35′38.2″ S, 72°58′43.5″ W), in November 2019. The samples were transported to the Natural Products Chemistry Laboratory, University of Concepción, Chile, under refrigerated conditions. Taxonomic species identification was performed according to what was described by Gonzaléz et al. [70]. Afterward, the macroalgae sample was washed with distilled water to remove epiphytic organisms, mineral particles, and salts. Finally, the samples were lyophilized and cut into pieces.

### 3.1. Polysaccharide Isolation

The extraction of polysaccharides from *C. bernabei* was carried out following the procedure reported by Parages et al. [36], with minor modifications [71]. Ten grams of lyophilized algae sample was treated with 500 mL of 85% ethanol, with constant stirring for 24 h at room temperature, for pigment removal. Afterward, the solid material was dried at 25 °C. The polysaccharide-rich fraction was extracted using distilled water as a solvent and a 1:50 (solid/liquid) ratio in a glass reactor vessel. The mixture was heated at 100 °C for 1 h under constant mechanical stirring. The mixture was allowed to settle down for 1 h and then centrifuged at 4500 rpm. The supernatant was removed and divided into two portions. One part was treated with cold ethanol (96% *v*/*v*) to recover the TPs fraction by precipitation. The second fraction was precipitated selectively with N-cetylpyridinium bromide (Cetavlon) 2% (*w*/*v*) to obtain the APs sample. Both polysaccharide fractions were purified by resuspending in 4 M NaCl solution and then dialyzed against a descendent concentration NaCl solution (from 2 to 1 M) for 12 h at 4 °C. Finally, the suspension was dialyzed against distilled water for 24 h and, lyophilized and weighed for the yield determination.

### 3.2. Polysaccharide Characterization

#### 3.2.1. Scanning Electron Microscopy (SEM) Analysis

The surface morphology of the extracted samples was analyzed by an ETEC autoscan SEM (Model U-1, University of Massachusetts; Worcester, MA, USA) at an accelerating voltage of 10 kV. The samples were fixed in a sample holder and sputtered with an Au layer using an Edwards S150 sputter coater (BOC Edwards, São Paulo, Brazil) before analysis.

#### 3.2.2. Elemental Analysis

The N elemental analysis was performed by a CHNS Elemental analyzer (varioMicro V1.6.1 (Elementar UK Ltd, Manchester, UK). The gas flow rate was 210 mL min^−1^ and 15 mL min^−1^ for He and O_2,_ respectively. The total protein content was determined by the formulae N (%) × 4.80 reported by Lourenço et al. [72].

#### 3.2.3. Fourier Transform Infrared Spectroscopy (FTIR) Analysis

The chemical structure of extracted samples was analyzed by Agilent Cary 360 FTIR-ATR (Agilent, Santa Clara, CA, USA) spectroscopy, in the range of 4000 to 500 cm^−1^, at a resolution of 4 cm^−1^ and 64 scans.

#### 3.2.4. Thermal Analysis

The thermogravimetric studies were performed with a thermobalance (Cahn-Ventron 2000, Agilent, Santa Clara, CA, USA) with a microprocessor, a temperature control unit, and thermal analysis data software. The samples (5–10 mg) were heated from 25 to 600 °C at a heating rate of 10 °C min^−1^ with a 50 mL min^−1^ nitrogen flow.

#### 3.2.5. Molecular Weight

GPC analysis was used to determine the sample weight-average (Mw), number-average (Mn) molecular weight, and polydispersity index (PI) [73]. The analyses were conducted in a GPC Waters chromatographer (Waters Corporation, Milford, MA, USA) equipped with an IR detector and three Waters Ultrahydrogel SEC columns (120–250–500). The eluent was 0.1 M sodium hydroxide and 0.05 M sodium acetate solution. Pullulan standards with different molecular weights (Mw 5–100 kDa) were used for MW calculation. The samples (100 μL; 1 mg mL^−1^) were dissolved in eluent and injected at 0.5 mL min^−1^. The column oven temperature was controlled at 30 °C. The data processing was performed with Empower GPC software.

#### 3.2.6. Determination of Sulfate Content

The sulfate contents of the polysaccharides were determined according to the protocol described by Therho and Hartiala [74], with some modifications by Wu et al. [75]. In brief, 1 mL polysaccharide solution (2 mg mL^−1^ in 0.5 N HCl) in a closed vial was hydrolyzed at 100 °C for 1 h. After evaporating the solvent in the vial, the dried hydrolyzed sample was sequentially added to 0.5 mL water, 2 mL 95% ethanol, 1 mL BaCl_2_ solution (0.1 mM), and 1.5 mL sodium rhodizonate solution (50 mg mL^−1^ containing 1 mg mL^−1^ L-ascorbic acid). After thoroughly mixing, the solution was allowed to stand in the dark at room temperature for 20 min, and absorbance at 520 nm was read by a UV/Vis spectrometer Multyskan Sky (Thermo Fisher, Waltham, MA, USA) The sulfate concentration was determined using a standard curve derived from known K_2_SO_4_ concentrations. The sulfate content of the sample was calculated using the following equation.

$$Sulfate\;content\;\left(\%\right)=\left(sulfate\;concentration\times total\;volume/sample\;amount\right)\times 100\%$$

#### 3.2.7. Monosaccharide Composition (GC/MS)

##### Hydrolysis and Derivatization

In a reaction vial (reagent-vial 3 mL, Thermo Fisher, Waltham, MA, USA), 2 mg of the polysaccharides extracted from *C. bernabei* were subjected to acid hydrolysis with 600 µL of HCl in 3 N methanol (Merck KGaA, Burlington, MA, USA), for 24 h at 80 °C. The solvent was then evaporated in a stream of nitrogen at 50 °C in an evaporator-concentrator (Stuart BlocK^®^ Heater, SBH200D/3, Merck KGaA, Burlington, MA, USA). Subsequently, to remove excess acid, the residue was washed three times with methanol and dried again. The samples were subjected to derivatization by silylation with 300 µL of Tri-Sil reagent (Pierce, Thermo) for 1 h at 80 °C. The reagent was then removed by a stream of nitrogen, reconstituted with 500 µL of hexane, and centrifuged for 15 min. The supernatant was filtered and re-evaporated, and reconstituted in 150 µL of hexane (LC-MS grade, Merck KGaA, Burlington, MA, USA) in a chromatography vial.

##### Gas Chromatography/Mass Spectrometry (GC-MS) Analysis

The fractions were characterized by Gas Chromatography-Mass Spectrometry (GC-MS) (Agilent 7890A, Santa Clara, California, USA), with an Agilent 5975C mass detector, using an HP5-MS type fused silica capillary column of 30 m, 0.25 mm inner diameter and 0.25 μm film thickness, under the following characteristics: temperature: 250 °C; detector (mass): 280 °C; furnace: initial 100 °C for 5 min, increasing by 8 °C min^−1^ up to 250 °C and maintained for 15 min [30]. The detector set in the scan mode ranged from 50 to 500 amu. The carrier gas flow (electronic degree helium) was at 1 mL min^−1^. The compound characterization was carried out by means of comparison with the NIST ^®^ database.

### 3.3. Biological Aactivity

#### 3.3.1. Antioxidant Activity

The antioxidant activity of TPs and APs was evaluated following the procedure of Brand Williams et al. [76], with slight modifications. Briefly, 20–500 μL solution of different concentrations of samples (0.0075–0.377 mg mL^−1^) or ascorbic acid (100 µg mL^−1^) were added to 500 µL 2,2-diphenylpicrylhydrazyl (DPPH) solution. The mixture was then incubated at 25 °C in the dark for 30 min. The absorbance was read at 517 nm by a UV-Vis spectrometer. The DPPH scavenging activity was calculated according to the following equation:

$$\mathrm{DPPH}\;\left(\%\right)=\frac{\left(\mathrm{Ac}-\mathrm{As}\right)}{\mathrm{Ac}\;\times \;100}$$

where, Ac is the absorbance of the control (100 µL of ethanol with 100 µL of the DPPH solution) at time zero, and As is the absorbance of the sample at the end of the reaction (30 min) at 517 nm. A calibration curve was made with different concentrations of Trolox^®^ from a stock of Trolox^®^ 1.268 mM. The dilutions were obtained at concentrations from 0 to 6 μM. All of the determinations were performed in triplicate (*n* = 3). The antioxidant capacity was expressed as a % of antioxidant activity.

The free radical scavenging activity of the TPs and APs was examined using ABTS radicals [77]. The ABTS radical cation (ABTS^•+^) was produced by reacting ABTS (2,20-azinobis(3-ethylbenzothiazoline-6-sulfonic acid diammonium salt) aqueous solution with 2.45 mM potassium persulfate at room temperature for 16 h. ABTS+ was dissolved in water and diluted with ethanol to an absorbance of 0.70 at 734 nm. Then, a 20–500 μL solution of native polysaccharide and commercial k-carrageenan (0.0075–0.377 mg mL^−1^) or ascorbic acid (100 µg mL^−1^) were added to 500 µL of 0.7 mM ABTS^•+^ solution. The solution was kept at room temperature for 30 min, and the absorbance was read at 734 nm. The ABTS^•+^ scavenging activity was calculated according to the following equation:

$${\mathrm{ABTS}}^{•+}\left(\%\right)=\frac{\left(\mathrm{Ac}-\mathrm{As}\right)}{\mathrm{Ac}\;\times \;100}$$

where, Ac is the absorbance of the control (0.5 mL of ethanol with 500 µL of the ABTS^•+^ solution) at time 0; As is the absorbance of the ABTS radical solution mixed with the sample after 30 min. A calibration curve was made with different concentrations of Trolox^®^ from a stock of Trolox^®^ 2.5 mM. Serial dilutions were obtained at concentrations of 5, 10, 15, and 20 μM. All determinations were performed in triplicate (*n* = 3). The antioxidant capacity was expressed as % antioxidant activity.

#### 3.3.2. Anticoagulant Activity

The anticoagulant activities of TPs and APs were carried out according to the method of Mourao et al. [78], using normal human plasma and plasma with vehicle dimethyl sulfoxide (DMSO) as the control. For this purpose, activated partial thromboplastin time (APTT) and prothrombin time (PT) coagulation assays were performed, using heparin as a reference. ACL TOP 350 equipment (Instrumentation Laboratory, MA, USA) was used for measurements. A range of concentrations from 10, 100, and 1000 µg mL^−1^ of polysaccharides was tested. The independent experiments were performed in triplicate n.

#### 3.3.3. Cell Viability in Tumor Lines

For the antitumor assay, HCT-116, MCF-7, and HL-60 cells were incubated at various concentrations of TPs and APs. Each cell line was incubated in a 96-well microplate for 72 h (37 °C, 5% CO_2_ in a humid atmosphere). Cell proliferation was estimated by MTT (3-(4,5-dimethylthiazol-2-yl)-2,5-diphenyltetrazolium bromide) [79]. Briefly, a volume of 10 μL of the MTT solution (5 mg mL^−1^ in saline solution plugged with phosphate) was added to each well, and the plates were incubated at 37 °C for 4 h. The yellow MTT tetrazolium salt was reduced by the mitochondrial dehydrogenase of metabolically viable cells in order to form insoluble purple formazan crystals that coukd be dissolved via the addition of acid-isopropanol (150 μL of 0.04N HCl—2-propanol) and measured spectrophotometrically at 550 nm (MicroPlate Reader 2001, Whittaker Bioproducts, IL, USA). Cell viability was expressed as the mean percentage of viable cells in comparison to untreated cells. Three samples for each concentration assayed in each experiment were included. The determinations were carried out in triplicate in independent experiments.

### 3.4. Statistical Analysis

To evaluate variation in the antioxidant activity of TPs and APs polysaccharides, a one-way analysis of variance (ANOVA) was performed. To compare the differences between each tumor lines treatment with polysaccharides, a one-way ANOVA was run, followed by a post hoc *Tukey’s* test. Prior to these analyses, the assumptions of normality and variance homogeneity were evaluated. All of the statistical analyses were performed with R version 3.2.3 (Core Team 2016). Differences were considered statistically significant when *p* < 0.05.

## 4. Conclusions

In this study, analyses showed that TPs and APs extracted from *Codium berteroi* were mainly composed of Gal, Glu, and other neutral sugars. Both polysaccharide fractions showed high thermal stability. The antioxidant activity showed that TPs had stronger antioxidant activity in vitro than APs. For TPs, it increases exponentially along with polymer concentration, which could be related to their chemical composition in terms of proteins, sugars, molecular weight, and degree of sulfation. However, in absolute values, it was lower than ascorbic acid used as a positive control. TPs and APs extracted from *C. bernabei* prolonged the TPPA and PT, thus inhibiting the blood coagulation of the samples. TPs showed higher anticoagulant activity than APs and might be a potential source of anticoagulant polysaccharides. Finally, the antitumor test in colorectal carcinoma (HTC-116) cell line, breast cancer (MCF-7), and human leukemia (HL-60) cell lines showed the cytotoxic effect of TPs (IC_50_ 760, 1118, and 540 µg mL^−1^) and APs (IC_50_ 1370, 1952, and 260 µg mL^−1^), respectively. The antiproliferative effect seems to depend on the cancer cell lines, and no clear relationship with structural parameters or antioxidant activity can be established in the present work. Further studies are in progress to clarify the mechanism and selectivity of the antitumor activity for both polysaccharides and their relationship with their physicochemical properties.

## Figures and Tables

**Table 1:** Physicochemical properties of *C. bernabei* polysaccharide fractions obtained in this work (TPs and Aps) and comparison with the physical chemical properties of other Codium macroalgae polysaccharides obtained from literature.

Parameter/Sample	TPs	APs	*Codium fragile* [5,13]	*Codium vermilara* [28,29]	*Codium decorticatum* [30,31,32]
Yield (wt%)	2.9	2.1	6.7	6.8	3.4
Sugar composition (mol%)					
Xylose	2.8	3.3	3.2	2	3.0
Glucose	31.1	19.3	4.8	1.9	5.1
Galactose	51.8	61.0	61.6	49.8	36.5
Fucose	1.2	2.1	tr ^a^	-	1.6
Mannose	7.4	6.1	3.9	3.6	2.6
Arabinose	5.7	8.2	23.1	44.7	48.8
Degree of sulfation (−SO_3_^2−^)	19.37	17.2	13.0	30.4	22.5
Protein content (wt%)	15.6	3.5	11.3	16.2	20.7
Mw (kDa)	17.9	14.9	11	66	
Mn (kDa)	8.9	9.1	-	-	-
Polydispersity Index	2.0	1.6	-	-	-

^a^ Percentage lower than 1% is given as trace (tr); Mw: Weight average molecular weight; Mn: Number average molecular weight.

**Table 2:** Thermogravimetric analysis of the polysaccharide fractions isolated from *C. bernabei* macroalgae.

Sample	Temperature (°C)			Weight Los (%)	Char (%)
	Onset	Peak	End		
TPs	37.1	66.3	164.3	8.2	36.2
	196.8	282.5	402.2	52.7	
APs	28.6	79.1	194.1	8.1	42.4
	208.6	271.7	393.5	37.2	

**Table 3:** Effect of TPs and APs polysaccharide fraction isolated from *Codium bernabei* on prothrombin time (PT) and thromboplastin time (APTT).

Samples	PT (s) *	APTT (s) *
Normal plasma pool	10.9 ± 0.2 A	25.6 ± 0.3 A
Normal plasma pool + DMSO	11.0 ± 0.1 A	26.4 ± 0.1 A
Normal plasma pool + TPs (1000 µg mL^−1^)	17.8 ± 1.3 B	>50
Normal plasma pool + TPs (100 µg mL^−1^)	10.9 ± 0.2 A	45.7 ± 2.5 B
Normal plasma pool + TPs (10 µg mL^−1^)	10.5 ± 0.1 A	26.9 ± 0.3 A
Normal plasma pool + APs (1000 µg mL^−1^)	10.4 ± 0.1 A	35.2 ± 0.1 B
Normal plasma pool + APs (100 µg mL^−1^)	10.7 ± 0.1 A	26.8 ± 0.1 A
Normal plasma pool + APs (10 µg mL^−1^)	10.7 ± 0.1 A	26.4 ± 0.2 A

* All clotting assays were performed in triplicate and were expressed as means ± SD. Different letters (A,B) indicate a significant difference between polysaccharides when compared at each concentration according to the (*p* < 0.001). Reference value for human plasma PT: 9.5–12.5 s, and human samples APTT: 25.4–36.9 s.
